# To total amount of activity….. and beyond: perspectives on measuring physical behavior

**DOI:** 10.3389/fpsyg.2013.00463

**Published:** 2013-07-22

**Authors:** Johannes B. J. Bussmann, Rita J. G. van den Berg-Emons

**Affiliations:** Department of Rehabilitation Medicine, Erasmus MC University Medical Center RotterdamRotterdam, Netherlands

**Keywords:** physical activity, measurement, validity, ambulatory monitoring, physical behavior

## Abstract

The aim of this paper is to describe and discuss some perspectives on definitions, constructs, and outcome parameters of physical behavior. The paper focuses on the following constructs: Physical activity and active lifestyle vs. sedentary behavior and sedentary lifestyle; Amount of physical activity vs. amount of walking; Detailed body posture and movement data vs. overall physical activity data; Behavioral context of activities; Quantity vs. quality; Physical behavior vs. physiological response. Subsequently, the following outcome parameters provided by data reduction procedures are discussed: Distribution of length of bouts; Variability in bout length; Time window; Intensity and intensity threshold. The overview indicates that physical behavior is a multi-dimensional construct, and it stresses the importance and relevance of constructs and parameters other than total amount of physical activity. It is concluded that the challenge for the future will be to determine which parameters are most relevant, valid and responsive. This is a matter for physical behavior researchers to consider, that is critical to multi-disciplinary collaboration.

## Introduction

Over the last few decades, the methods used to objectively assess a person's behavior in terms of body postures (e.g., sitting, standing), body movements (e.g., walking, cycling), and/or daily activities (e.g., sports, gardening) in a daily life setting have improved considerably. Devices have become smaller, power consumption requirements have decreased, data storage capacity has increased, and innovative, integrated sensors have been developed. From the beginning, outcome variables related to this type of behavior mainly focused on amount and volume parameters, such as number of steps, volume of physical activity as expressed by total number of counts, and total energy expenditure. These developments and outcome variables have contributed to a better understanding of daily behavior and a more accepted role of it in research and clinical practice.

However, at the same time, the development of knowledge within this area is threatened by some theoretical and methodological issues. Firstly, vagueness and variability exist in terminology, concepts and definition of behavior related to body postures, movements and daily activities. An example of this is the term *physical activity*. This term is most common in literature, and mostly defined as any bodily movement produced by skeletal muscles that requires energy expenditure (Caspersen et al., 1985). However, physical activity as defined in this way does not cover all aspects of behavior that can be relevant (e.g., body postures as sitting and standing), and therefore cannot be used as umbrella term.

At the same time, the term is defined and used in ways that significantly differs from the definition stated above, as stated by Pettee Gabriel et al. (2012). Therefore, we will use and propose in this paper the term *physical behavior* as umbrella term, which includes the behavior of a person in terms of body postures, movements, and/or daily activities in his/her own environment.

Secondly, the importance of well-selected outcome measures is not always fully recognized. For example, physical behavior is not only characterized by amount or volume, but also by other aspects, as illustrated below with an example from research we were involved in. In 2004 Garssen et al. performed a study on effects of training in severely fatigued patients with Guillain Barre Syndrome (Garssen et al., 2004). It was assumed by doctors and therapists that this group had a low level of physical fitness, that they were hypoactive and that they had a lot of problems with functioning and participation in daily life. These assumptions were indeed confirmed, with the exception of the assumption on hypoactivity. No significant difference in amount of being physically active (i.e., time spent in walking, cycling, running, etc.) was found with healthy controls, and no significant effects of an exercise program on this parameter were observed. In the discussion section it was concluded: “In contrast with most physiologic and subjective variables, objectively measured daily physical activity using the Rotterdam Activity Monitor did not show any significant increase in activity. This may suggest that changing the level of daily physical activity is not an important adaptation strategy in these fatigued patients.” Although it is uncertain that this conclusion is false, it is justified to put some question marks behind it. Did we really focus on the right aspect of physical behavior?

Most overview or review papers so far have focused on characteristics of, and differences between, techniques, and devices. The aim of this paper is to describe and discuss some perspectives on measuring physical behavior, with a distinction between different constructs of physical behavior, and different outcome variables resulting from data reduction procedures. The paper does not pretend to give a complete overview of literature, but aims to demonstrate by examples that physical behavior is more than total amount of activity.

## Constructs of physical behavior

### Physical activity and active lifestyle vs. sedentary behavior and sedentary lifestyle

As already stated, physical activity can be defined as any bodily movement produced by skeletal muscles that requires energy expenditure (Caspersen et al., 1985). The volume of physical activity is mostly expressed by the number of activity counts per time period, which depends on the amount and intensity of movement. From these counts, energy expenditure can be estimated. When the type, amount and/or intensity of physical activity or energy expenditure over longer periods (e.g., a week) exceeds defined guidelines, this behavioral pattern can be characterized as an “active lifestyle.” So far, most studies that aimed at (improving) health have focused on measuring the volume of physical activity or energy expenditure from this perspective. However, recent studies showed the relevance of sedentary behavior. The term sedentary is related to the Latin word “sedere” (to sit) and defined as “any waking sitting or lying behavior with low energy expenditure” (Wilmot et al., 2012). However, also other definitions exist, such as “sitting without being otherwise active” (Owen et al., 2011), or “a distinct class of activities that require low levels of energy expenditure and involve sitting during commuting, in the workplace and the domestic environment, and during leisure” (Thorp et al., 2011). These definitions broadly fit with—but are not similar to—the commonly used criterion of 1–1.5 metabolic equivalent units (MET's; multiples of basal metabolic rate) (Wilmot et al., 2012).

Sedentary behavior is not just the counterpart of physical activity (Lord et al., 2011; Owen et al., 2011; Wilmot et al., 2012). For example, a person cannot be “active” and “sedentary” at the same moment, but he/she can have an “active lifestyle” from the perspective of physical activity or energy expenditure, and simultaneously be characterized by having a sedentary lifestyle because of long periods of sitting or reclining with low levels of energy expenditure. That sedentary behavior patterns are different from just low levels of physical activity is supported by several studies, that show the active lifestyle-independent relationship between sedentary behavior and disease, health markers, and mortality (Proper et al., 2011; Thorp et al., 2011; Wilmot et al., 2012). Thus, the literature indicates that health-related research must not only focus on physical activity and its guidelines, but also on sedentary behavior.

### Amount of physical activity vs. amount of walking

So far, many studies have specifically focused on amount aspects of walking, including number of steps, distance walked, and walking time. Step counting was one of the first, widespread applications of activity monitoring. The underlying idea of step counting is that walking is the most important modality of physical activity and that it is a major contributor to activity-related energy expenditure (Bravata et al., 2007). This point of view is also reflected in studies including public health recommendations in terms of steps/day (Tudor-Locke and Bassett, 2004). Walking parameters like the number of steps can be relevant from certain perspectives, but besides methodological problems [e.g., in low walking speeds (Feito et al., 2012) or distorted walking (Mudge et al., 2007)], the extrapolation to total level of daily physical activity has to be done with care. That being physically active is not similar to walking is also shown by data of our department from a large data set of healthy, Dutch control subjects who were measured with the Vitaport Activity Monitor (TEMEC Instruments, Kerkrade, The Netherlands) (Bussmann et al., 2001), a device that allows detailed body posture and movement detection. These data showed that overall in this population walking duration only contributes for 75% to the duration of being active and, besides that, there is a large inter-individual range (43–98%) (unpublished observation). Therefore, it can be concluded that number of steps or walking duration is a questionable-valid estimator of time being active, and that a considerable underestimation may occur.

### Detailed body posture and movement data vs. overall physical activity data

Most accelerometer-based wearable monitors are based on the principle of movement counts from a single sensor, with the number of counts depending on the amount and intensity of movements. A limitation of this approach is that no distinction can be (easily) made between different postures, movements and daily activities. As described in the preceding paragraph, sedentary behavior, can be approached from the perspective of energy expenditure and from the perspective of body postures as sitting. The first perspective needs techniques that validly measure energy expenditure/MET's, the second one requires body posture detection. The ActivePAL device (PAL technologies, Glasgow, UK) is currently considered a reference method for discriminating sitting, standing, and ambulation. The potential relevance of detailed body posture and movement data has been shown by Hamilton et al. (2007), who have provided evidence (based on electromyography and lipoprotein lipase activity) that sitting and standing are physiologically different. Detailed posture and movement data can also be used to improve the estimation of energy expenditure in daily life (Bonomi et al., 2009). An example from another perspective is a study by Cumming et al. (2011), who reported favorable effects of early mobilization, with lying in bed being considerably different from a mobilization point of view than sitting out of bed. These examples express some potential benefits of data on specific postures and movements.

### Behavioral context of activities

Most studies and devices focus on physical behavior over the whole day, for example the number of steps per day. It might be, however, that not the total quantity (amount or volume) is of interest, but the quantity performed within a specific behavioral context of activities (Giles-Corti et al., 2005). Thus, the target (Giles-Corti et al., 2005) or domain (Healy et al., 2011a) of that activity (e.g., shopping, watching television) as well as the setting or physical environment (e.g., walking indoors, outdoors) should then be considered. This issue is well described in a paper of Giles-Corti et al. (2005), and an example is a study of Duncan et al. (2009) which studied the relationship between built environment and walking outdoors (defined as transport-related physical activity and recreational walking). In such a study, not all walking periods are of interest, but mainly the periods of walking performed outdoors. The behavioral context is mostly assessed by observation or diaries and questionnaires, but future technological developments will also allow registration in other ways, such as GPS (Duncan et al., 2009) and miniature camera systems. Examples and discussion of combining the assessment of physical behavior and context variables using e-dairies in everyday life can be found in literature (Bussmann, 2013; Ebner-Priemer et al., 2013; Kanning, 2013).

### Quantity vs. quality

Physical behavior not only concerns amount and volume (quantity), it's also about the way activities are performed (or quality). It might be that diseases or interventions do not affect quantity, but do affect quality. Every posture, movement, or physical activity has its own quality aspects. For example, in walking symmetry, stability, spatio-temporal parameters, and walking speed are examples of quality parameters. A good example of this quantity-quality issue are recent studies by de Groot et al. and Vissers et al. on the effects of osteoartrosis and total hip artroplasty on physical behavior (de Groot et al., 2008; Vissers et al., 2011). When quantity parameters were analysed—such as time spent walking and number of sit-to-stand transitions—no differences were found pre-surgery between patients and healthy controls, and no effects were found from pre- to post-surgery. When quality data were analysed—such as walking speed and speed of rising from a chair—significant differences and effects of treatment were found. So apparently, diseases and surgery did influence physical behavior, but not quantitative aspects of physical behavior.

### Physical behavior vs. physiological response

Many accelerometers aim to estimate energy expenditure, which results from, but is no part of physical behavior. With the focus on energy expenditure, movement counts are converted to kilocalories, mostly with gender, weight, height, and age taken into account. However, movement efficiency (the ratio of external work performed compared to the internal energy expended to do the work) is generally not considered and, especially in disabled people, movement efficiency might considerably differ between persons and groups. As a result, the relationship between e.g., movement counts and energy expenditure will strongly vary.

For example, persons walking with a lower limb prosthesis have been shown to have a similar activity count as healthy controls when walking at a fixed speed, but the physiological response (expressed by heart rate and oxygen uptake) was significantly higher in amputees (Bussmann et al., 2004b). In such cases, accelerometry will underestimate the actual energy expenditure. From another perspective, this phenomenon was also found in another study by our group (Bussmann et al., 2004a, 2008): compared with healthy controls, people with an amputation were shown to walk less and at a lower walking speed, but the heart rate during walking was not significantly different, demonstrating the conceptual difference between physical behavior parameters and its physiological responses. It can be concluded that—especially in persons or groups that differ in movement efficiency—physical activity and the associated physiological response are different constructs, but both are important to assess.

## Physical behavior outcome variables resulting from data reduction procedures

The previous paragraph focused on different conceptual constructs of physical behavior. However, the same construct can be operationalized in different ways, and different constructs similarly. Actually, it is an issue of data processing. In the literature, amount, and volume of physical behavior outcome variables are usually presented in terms of duration and frequency, such as the mean count per minute, the total number of steps, and the number of sit-to-stand transitions. It can be questioned whether these overall outcome variables are sufficiently relevant, valid, and responsive in all conditions. In this paragraph we will discuss some additional methods of data processing, aiming at bout length and frequency, variability in bout length, the time window, and intensity and thresholds.

### Bout length and frequency

**Figure 1:**
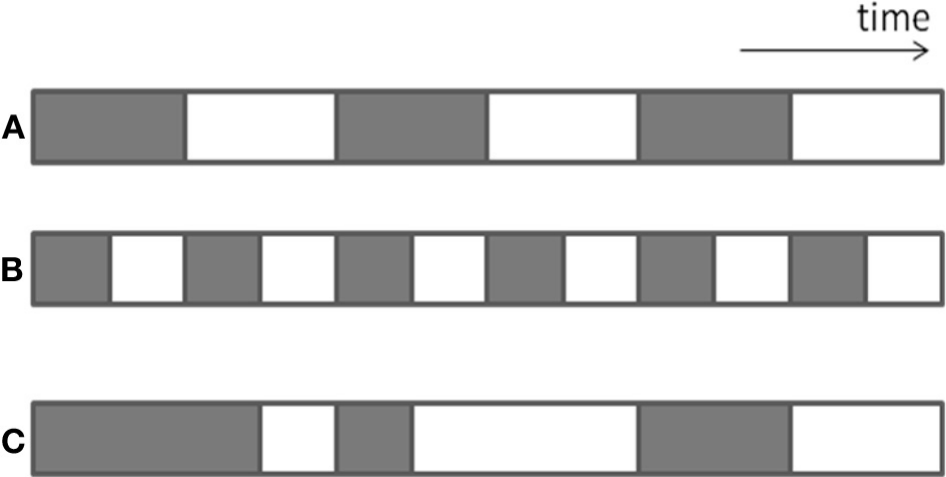
**Graphical representation of differences in distribution of length of bouts and variability in bout length**. The gray bars indicate sedentary behavior (sitting).

**Figure 2:**
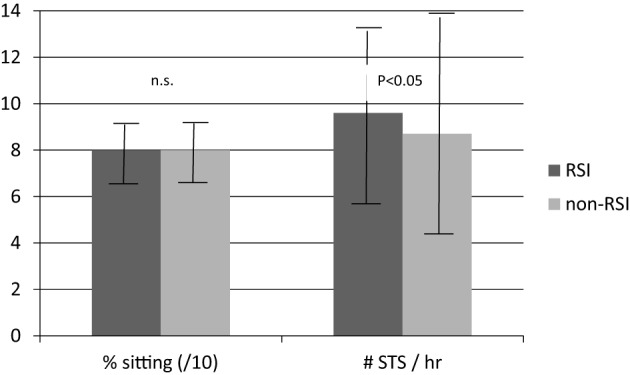
**Mean and SD of percentage sitting (for graphical reasons divided by 10) and number of sit-to-stand transitions per hour during working hours in computer workers with and without RSI**.

The same amount and volume of body postures and movements and daily activities can be achieved by many short bouts interspersed throughout the day or from few long bouts (Figure 1; bars A,B). For example, 60 min of walking during a regular day can be composed of 30 walking bouts of 2 min, or two walking bouts of 30 min. The importance of bout length is described in literature. For example, a study of Healy et al. (2011b) suggests that besides reducing sedentary time, breaking up sedentary time may be beneficial for cardiovascular disease risk. Another example is provided by Chastin et al. (2010) who showed that persons with Parkinson Disease (PD) did not differ from healthy subjects in the total amount of sedentary time, but did significantly differ in distribution parameters: subjects with PD had longer continuous periods of sedentary behavior than their healthy comparison subjects. In a study of our group (Keijzer-Oster et al., unpublished data) we explored the physical behavior of computer workers with and without Repetitive Strain Injury (RSI). Data showed that the two subgroups did not differ in overall amount of sitting, but that there was a significant difference in number of breaks in sitting, expressed by the number of sit-to-stand transitions (see Figure 2). In contrast to the expectations, subjects with RSI had a larger number of STS transitions. Generally, these results indicate that in some cases no effect is found on amount or volume measures, whereas these are found in the area of distribution.

### Variability in bout length

The same total amount can be built up from similar-length bouts (i.e., of the same duration, Figure 1; bar B), or from bouts with variable length (Figure 1; bar C). There are many physiological processes in human functioning in which variability is considered to be important, for example as a measure of capability to adapt to different circumstances. For example, Madeleine et al. (2008) showed that workers with pain and workers with less work experience had less variable movement patterns than those without pain or with more experience. On the other hand, too much variability may be disadvantageous; e.g., patients with PD are not able to walk with a consistent gait pattern, and this increased variability is associated with PD symptoms and fall risk (Weiss et al., 2011). To date, studies have rarely focused on variability analysis of activities of interest, but it can be hypothesized that variability in bout length for specific activities also represents the ability to adapt and/or consequences of diseases on physical behavior. Similarly, variability in other aspects of physical behavior (e.g., walking speed, daily activity counts) and in the physiological responses resulting from it (such as heart rate) might express the capacity to adapt.

### Time window

In studies of physical behavior, data are generally averaged over the whole measurement period. By such analyses it might be that effects are averaged out. An example of this phenomenon is a study by Rochester et al. (2006). They compared volume measures such as amount of time walking, amount of time standing, and number of walk periods in subjects with PD vs. healthy control subjects. Whole-day analyses showed no significant differences between these groups. When data were expressed on an hourly basis, however, different patterns were found. This means that the relevance of the outcome variables depends on the time window of analyses. Similarly, this might be the case between weekend and work days, with possibly no differences on a weekly basis, but significant differences when a distinction is made between work and weekend days. Therefore, the time window of analyses should be carefully chosen before starting a study.

### Intensity and intensity threshold

In many cases the overall activity count or energy expenditure is assessed. However, the same overall activity count can result from long periods of low-intensity physical activity or a short period of vigorous physical activity. From a health perspective, the physiological effect of these two examples will be considerably different. Therefore, in many studies and instruments, the data are not only presented as, for example, mean MET score, but also as minutes in different intensity or MET categories (e.g., light, moderate, vigorous; e.g., Ekelund et al., 2011).

One example is a study of Janz et al. (2006), which showed that the number of minutes above a certain intensity level is related to femoral neck bone strength in children. In the same line of reasoning, Duvivier et al. (2013) concluded that given constant energy expenditure, reducing inactivity by increasing the time spent walking/standing is more effective than one hour of physical exercise, from the perspective of insulin sensitivity and plasma lipids.

## Discussion

In this overview the umbrella term “physical behavior” was purposely used. Although we realize that physical activity is a more familiar construct, we also feel that the term is confusing and that it does not logically and semantically cover all its underlying constructs. Others already attempted to create a conceptual framework of physical activity, e.g., Pettee Gabriel et al. (2012). They also recognize the umbrella concept of (physical) behavior. However, their model is strongly based on the distinction between two types of behavior (physical activity and sedentary), whereas we feel that physical behavior has much more relevant descriptors and components. In agreement with Pettee Gabriel et al., we feel that (physical) behavior does not include the physiological responses resulting from it, although they will be strongly related to each other.

As stated in the introduction section, the issues and cases described and discussed in this paper are examples to illustrate the central message that physical behavior is more than amount and volume, and that failure to find an effect or differences does not mean that there are no effects on physical behavior. We realize that in this paper literature is not extensively and systematically discussed, and that the given examples are arbitrary. A next step might be a systematic and in-depth review on some of the topics that are discussed in the current paper.

From our perspective we feel that the challenge for the future will be to determine which parameters are clinically relevant, valid, and responsive. There will be no general answer on this question: the parameter of interest will necessarily depend on the purpose of measurement. As formulated by Terwee et al. (2011): “… for measuring physical activity as a risk factor for developing osteoarthritis an instrument should measure the mechanical load [on the joints]…. for measuring physical activity as a protective factor against functional decline, an instrument should measure frequency and duration of recreational activities such as walking and cycling.” We feel that in several cases a device is used and data are presented just because that device was available and has a specific parameter as main outcome variable. We therefore strongly recommend a clear analysis of the aim of the study and measurement and the component of physical behavior of interest, in line with the reasoning of e.g., Warren et al. (2010) and Clanchy et al. (2011).

If possible, that aim must be hypothesis-driven and embedded in current state of knowledge; random “data fishing” must be avoided as much as possible. As a result, we also strongly recommend that studies focus on underlying mechanisms. Of course, RCT's focusing on physical behavior and/or including measurement of physical behavior will be important, but understanding the role of physical behavior in the problem of interest and its determinants are at least equally important.

Stating that defining relevant, responsive and valid outcome variables must be based on a good question is a somewhat simplified way of reasoning. Many factors and people play a role in defining the question and outcome variables produced by data reduction procedures. First of all, people with practical knowledge and experience, such as doctors and therapists, are essential. Technicians and data analysts are needed for developing usable hardware and software that have relevant outcome variables as output. Researchers are necessary for e.g., methodologically testing outcome variables produced by data reduction procedures and for integrating research projects in current scientific knowledge. Together with other disciplines they can introduce and test new theories and models, possibly originating from other areas and disciplines. From our point of view it's a choice between “following the status quo,” and hoping to hit the target with luck, or a coordinated, focused and multi-disciplinary action.

## Recommendations

To improve the research on physical behavior, we recommend for new studies:

1. To be aware of the “state of the art” of physical behavior measurement and outcome parameters;
2. To make a clear link between (clinical) problem and (relevant) behavior parameters;
3. To specify in the research question the aspects of physical behavior at interest;
4. To describe and discuss the selection of parameters in the paper;
5. To describe in detail the measurement settings and applied data reduction procedures;
6. To select devices meeting the research question and selected outcomes, not vice versa;
7. To use outcomes that are tested in focused and sound validation studies;
8. To consider the added value of (simultaneous) acquisition of other data, such as physiological and psychological parameters, and context;
9. To be eager to be innovative and inventive;

### Conflict of interest statement

The authors declare that the research was conducted in the absence of any commercial or financial relationships that could be construed as a potential conflict of interest.
